# Supplementary material for: Genetics and Genomics of SOST: Functional Analysis of Variants and Genomic Regulation in Osteoblasts
Source: Int J Mol Sci. 2021 Jan 6;22(2):489. doi: 10.3390/ijms22020489 (PMC7825314; doi:10.3390/ijms22020489)
Supplement: Supplementary file 1 [file ijms-22-00489-s001.pdf]

International Journal of *Molecular Sciences*

MDPI

Article

Genetics and Genomics of *SOST*: Functional Analysis of Variants and Genomic Regulation in Osteoblasts

Núria Martínez-Gil [1], Neus Roca-Ayats [1], Mónica Cozar [1], Natàlia Garcia-Giralt [2], Diana Ovejero [2], Xavier Nogués [2], Daniel Grinberg [1] and Susanna Balcells [1,*]

[1] Department of Genetics, Microbiology and Statistics, Faculty of Biology, CIBERER, IBUB, IRSJD, Universitat de Barcelona, 08028 Barcelona, Spain; airun91@gmail.com (N.M.-G.); neroca@clinic.cat (N.R.-A.);
monicacozar@ub.edu (M.C.); dgrinberg@ub.edu (D.G.)

[2] Musculoskeletal Research Group, Centro de Investigación Biomédica en Red en Fragilidad y Envejecimiento Saludable (CIBERFES), ISCIII, IMIM (Hospital del Mar Medical Research Institute), 08003 Barcelona, Spain;
ngarcia@imim.es (N.G.-G.); dovejero@imim.es (D.O.); xnogues@parcdesalutmar.cat (X.N.)

* Correspondence: dgrinberg@ub.edu (D.G.); sbalcells@ub.edu (S.B.)

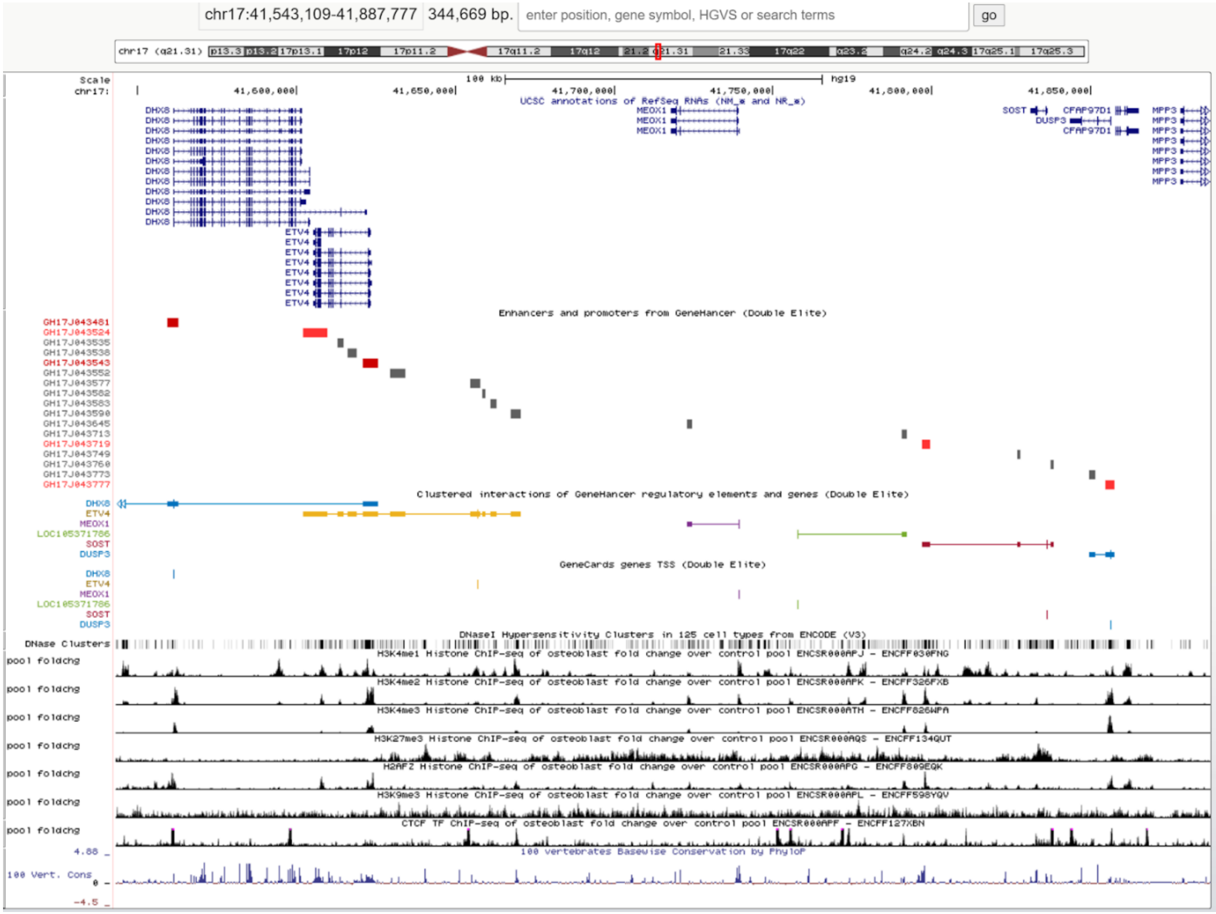

Supplementary Figure S1. Schema of *SOST* genomic region from the UCSC genome browser with the following tracks (downwards): In red, the promoter regions and in grey the enhancer regions according to GeneHancer track. In different colors, the interaction regions according to GeneHancer track and the TSS for each gene. In black, osteoblast ChIP-seq profiles of H3K4me1, H3K4me3, H3K4me2, CTCF, H3K27ac, H3K9me3 and H2A.Z from ENCODE. In blue, vertebrate conservation.

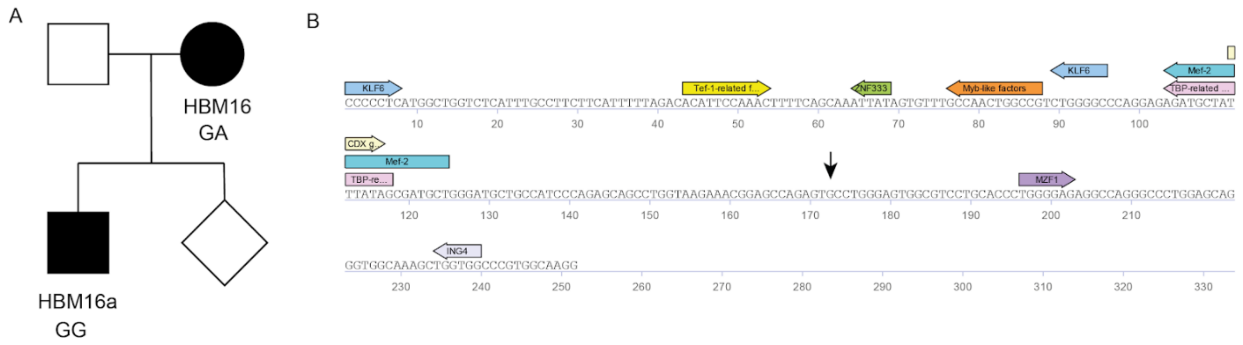

Supplementry Figure S2. Study of the rs552004150 variant. (**A**) HBM16 family pedigree, in which 2 individuals (HBM16 and HBM16a; filled symbols) present with the HBM phenotype. The SNP genotype is indicated below the symbols; family members with no information on the bone density phenotype are displayed in white. (**B**) TRANSFAC result of the *ECR5* region. The position of the variant is indicated with a vertical arrow.

Supplementary Table S1. GTEx eQTL data for all variants found in the resequencing of *SOST.*

SNP	Gene	Tissue
rs1237278	*SOST*	artery-tibial, aorta, coronary; lung; heart-atrial appendage; brain-cortex
	MPP3	thyroid
	NAG3	skin-sun exposed
rs851058	*SOST*	artery-tibial, aorta, coronary; lung; heart-atrial appendage; thyroid
	C17orf105	brain-cerebellum
	CD300LG	muscle-skeletal
	DUSP3	cells-cultured fibroblasts; whole blood; esophagus-mucosa
	MPP2	skin-sun and not sun exposed
	MPP3	cells-cultured fibroblasts
	NAGS	lung; skin sun exposed
rs10534024	*SOST*	artery-tibial, aorta, coronary; lung; heart-atrial appendage; brain-cortex, nerve-tibial
	MPP3	thyroid; testis
rs17882143	*DBF4B*	brain-caudate; thyroid
	RPL27	artery-aorta
rs17886183	*DUSP3*	whole blood
rs17881550	*SOST*	artery-tibial, aorta, coronary; heart-atrial appendage; lung; thyroid; nerve-tibial
	AOC2	lung
	C17orf105	brain-cerebellum

CD300LG	muscle-skeletal
DUSP3	esophagus-mucosa
MPP2	skin-sun and not sun exposed
NAGS	skin-sun exposed; lung

Supplementary Table S2. In silico predictions for the 3 SNPs in *SOST*.

SNP	Prediction	Allele	
rs17885799	ESE	C	Two new Exonic Splicing Enhancer (ESE)
-	-	-	miRNA
rs17883310	miRNA	C	hsa-miR-1915-3p<br>hsa-miR-3141<br>hsa-miR-4685
rs17886183	miRNA	A	hsa-miR-5583-3p

ESE: New Exonic Splicing Enhancer predicted. miRNA: Differences in the miRNA binding site depending on the allele.
